# Exploring the Association Between Heart Rate Control and Rehospitalization: A Real-World Analysis of Patients Hospitalized with Heart Failure with Reduced Ejection Fraction

**DOI:** 10.1007/s40801-024-00436-z

**Published:** 2024-08-01

**Authors:** Freny Vaghaiwalla Mody, Ravi K. Goyal, Mayank Ajmera, Keith L. Davis, Alpesh N. Amin

**Affiliations:** 1 Division of Cardiology 111E, Department of Medicine, Veterans Affairs Greater Los Angeles HCS, and the Department of Medicine at Ronald, Reagan University of California Medical Center, Los Angeles (UCLA) at the David Geffen School of Medicine at UCLA, 11301 Wilshire Blvd, Los Angeles, CA 90073 USA; 2 RTI Health Solutions, 3040 East Cornwallis Road, Research Triangle Park, NC 27709-2194 USA; 3 University of California, 101 The City Drive South, Building 26, Room 1000, Irvine, CA 92868 USA

## Abstract

**Background:**

In patients with heart failure with reduced ejection fraction (HFrEF), lower discharge heart rate (HR) is known to be associated with better outcomes. However, the effect of HR control on patient outcomes, and the demographic and clinical determinants of this association, are not well documented.

**Objectives:**

The purpose of this work was to evaluate the association between the HR control and the risk of post-discharge rehospitalization in patients hospitalized with HFrEF.

**Methods:**

Data were collected using a retrospective medical record review in the USA. Reduction in HR between admission and discharge (“HR control”) defined the primary exposure, categorized as no reduction, > 0 to < 20% reduction, and ≥ 20% reduction. Time to first rehospitalization in the post-discharge follow-up defined the study outcome and was analyzed using multivariable Cox regression modeling.

**Results:**

A total of 1002 patients were analyzed (median age, 63 years; median follow-up duration, 24.2 months). At admission, 59.1% received beta-blockers, 57.4% received diuretics, and 47.5% received angiotensin-converting enzyme (ACE) inhibitors. Most patients (90.5%) achieved some HR control (38.4% achieved > 0 to < 20% reduction, and 52% achieved ≥ 20% reduction). Approximately 39% were rehospitalized during the follow-up (14% within 30 days). In multivariable analysis, patients with > 0 to < 20% reduction in HR had a 39% lower risk of rehospitalization [hazard ratio 0.61; 95% confidence interval (CI) 0.43–0.85]; patients with ≥ 20% reduction in HR had a 38% lower rehospitalization risk (hazard ratio 0.62; 95% CI 0.45–0.87) than those with no HR reduction.

**Conclusions:**

Reduction in HR between admission and discharge was associated with reduced risk for rehospitalization. Findings indicate HR control as an important goal in the management of patients hospitalized for HFrEF.

**Supplementary Information:**

The online version contains supplementary material available at 10.1007/s40801-024-00436-z.

## Key Points

**Table:** 

We conducted a large real-world study of patients hospitalized for heart failure with reduced ejection fraction (HFrEF) in the USA and examined the link between their heart rate (HR) control and the risk of future rehospitalization.
Our analysis of 1002 patients showed that patients whose HR was reduced during the index hospitalization were at a significantly lower risk of getting hospitalized again in the near future (within 2 years) as compared with patients who did not achieve HR reduction.

## Introduction

Approximately 6.7 million adults in the USA have heart failure (HF), with an incidence of nearly 21 per 1000 adults older than 65 years and an age-adjusted mortality rate of 92 per 100,000 [1]. In the > 65 years age group, HF is the leading cause of rehospitalization [2]. More than 1 million hospitalizations across all age groups annually are estimated to cost the US health system $30 billion [1, 3, 4].

The evidence-based clinical approach to HF treatment recommended in the American College of Cardiology/American Heart Association guidelines has led to a reduction in mortality over time, mostly related to the use of combinations of angiotensin-converting enzyme (ACE) inhibitors, beta blockers (BB), and mineralocorticoid receptor antagonists [5–8]. The benefit of heart rate (HR) reduction in the management of HF has been recognized for at least 2 decades [9]. HR has been retrospectively shown to be an independent modifiable risk factor in patients with HF with reduced ejection fraction (HFrEF); reducing HR is associated with decreased morbidity and mortality, reduced costs of rehospitalization, and improvements in health-related quality of life [10–16]. However, despite the high healthcare burden of HFrEF, evidence on the extent of HR control and the impact of HR control on the risk of rehospitalization during post-discharge follow-up is limited. The objective of this retrospective medical record review was to describe the treatment patterns and evaluate the association between HR control and the risk of rehospitalization among patients hospitalized with HFrEF.

## Materials and Methods

### Study Design

This was a retrospective, noninterventional study based on abstractions of medical records of US patients hospitalized with acute HFrEF between 1 July 2013 and 30 June 2016 (index hospitalization). Patients who met eligibility criteria were selected by participating physicians who regularly treat HF.

### Physicians

Participating physicians were practicing in the USA at teaching and nonteaching hospitals, as well as at freestanding community clinics, as one of the following kinds of specialists: cardiologist; hospitalist; or specialist in internal medicine, family medicine, critical care, emergency medicine, or geriatric medicine. Physicians were required to (1) manage at least five patients with HF in an inpatient setting in a typical month, (2) be practicing in an inpatient setting (hospital or clinic) at least part-time, and (3) have had at least 3 years in clinical practice.

### Patients

Patient eligibility was determined using hospital admission data. Patients aged ≥ 18 years at index admission were included for medical record abstraction if they had a primary diagnosis of HF with left ventricular ejection fraction (LVEF) ≤ 35% (diagnosed via an echocardiogram at time of admission); had at least one of the following: (1) an intravenous diuretic administered, (2) brain natriuretic peptide (BNP) > 200 pg/mL, or (3) N-terminal (NT) pro-BNP > 600 pg/mL; and were discharged alive from the index hospitalization. Patients had at least 15 months of potential follow-up (unless death occurred earlier). Patients were excluded if they had a history of heart transplant or a major heart-related surgery in the 12-month period before index hospitalization. Patients who had participated in a clinical trial related to HF treatment at any time during the study period were excluded, as were patients with a history of severe valvular heart disease (e.g., aortic stenosis, mitral regurgitation) or any history of coronary artery bypass graft or valve surgery.

### Data Abstraction

Physicians abstracted data from individual patient medical records and entered deidentified data into a web-based electronic data collection form via a secure web-based data collection portal. The web-based data collection form included internal validity checks, with immediate error alerts if out-of-range values were entered. Additionally, use of the web-based system promoted completeness of data collection; completion of mandatory fields was required before physicians were permitted to progress to subsequent data fields.

### Study Measures

#### Baseline Demographics and Treatments

Baseline data from the 12-month period before the index hospitalization were identified, including patient demographics, comorbidities and risk factors, use of medications and devices for the management of HF, and history of HF-related hospitalization. For baseline comorbidity burden, a Charlson Comorbidity Index (CCI) score was calculated. Clinical and functional measures included LVEF and New York Heart Association (NYHA) functional class at admission, blood pressure (BP), and HR at admission and discharge. Treatment characteristics at admission, during admission, and at discharge were collected, including medication use and medication dose [e.g., BBs, ACE inhibitors, angiotensin receptor blockers (ARBs)], treatment modifications (e.g., dose reductions, discontinuations) and reasons for dose reductions, and use of medical devices (e.g., implantable cardioverter defibrillator, cardiac resynchronization therapy).

#### Primary Exposure

The primary exposure, HR control, was defined as percent reduction in HR between index admission and discharge and was categorized as no reduction, 0% < reduction < 20%, and ≥ 20% reduction; the cutoffs were driven by statistical consideration to ensure sufficiently large groups with reasonable clinical significance. The data on HR were collected for the first measurement taken prior to initiation of any treatment (in the hospital or in the emergency department); for discharge, it was the measure based on last set of vitals taken before discharge as usually recorded in the EHR system.

#### Outcome

Data on rehospitalizations occurring during the observed follow-up period after discharge from the index hospitalization were recorded. The outcome variable was defined as time to first rehospitalization at any time after discharge from the index hospitalization. Rate of rehospitalization within 30 days was also documented.

### Statistical Analyses

All study measures were descriptively analyzed. Time to rehospitalization was assessed using the Kaplan–Meier method, and median time to event was reported along with 95% confidence intervals, where estimable. Multivariable Cox proportional hazards models were used to assess the associations between HR control (percent reduction in HR between admission and discharge) and the risk of rehospitalization. Baseline patient characteristics were appropriately controlled for in all models. Patients with hospitalization of HF are generally expected to be on diuretics; however, our data included a fraction of patients with no diuretic use during the index hospitalization. Therefore, we performed a sensitivity analysis among patients who received treatment with a diuretic at any time during the index hospitalization to confirm that patients not on diuretics were not mis-enrolled and that those on diuretics behaved similarly to the overall cohort with respect to outcomes for the same level of HR control. All analyses were performed using SAS^®^ statistical software version 9.4 or later (SAS Institute, Cary, NC).

## Results

### Physicians and Patients

A total of 180 physicians with a mean [standard deviation (SD)] caseload of 79.3 (89.8) HF patients per month participated in the medical record abstraction. Physicians were predominantly cardiologists (57.8%) and had a mean (SD) of 13.9 (7.1) years of experience treating HF. They were primarily located in the Northeast (35.6%) and South (30.6%) and were hospital based (teaching hospital, 40.6%; nonteaching hospital, 42.2%) (Supplementary Table 1).

Overall, data were abstracted for 1002 eligible patients hospitalized with HFrEF during the study period. The median age was 63 years (range 20–104), and 62.7% of patients were male (Table 1). Median HR at admission was 90 bpm [interquartile range (IQR) 80–102], median systolic BP was 142 mmHg (IQR 118–160), and median diastolic BP was 82.5 mmHg (IQR 68–94). The median length of follow-up was 24.2 months (IQR 15.8–37.9). Of note, 37 patients (3.7%) died before the end of follow-up; 24 of these died from HF or HF-related complications.

**Table 1:** Patient characteristics

Characteristic	Patients (*N* = 1002)
Age at index date,^a^ years	
Mean (SD)	62.5 (13.3)
Median (range)	63 (20, 104)
Sex, *n* (%)	
Female	374 (37.3)
Male	628 (62.7)
Race/ethnicity, *n* (%)	
Non-Hispanic white	512 (51.1)
Non-Hispanic Black or African American	253 (25.3)
Hispanic or Latino	123 (12.3)
Asian	69 (6.9)
Other or don’t know	45 (4.5)
Insurance type at index date,^a^ *n* (%)	
Medicare	412 (41.1)
Private	279 (27.9)
Medicaid	144 (14.4)
Other or don’t know	167 (16.7)
Smoking status at index date,^a^ *n* (%)	
Never smoked	740 (73.9)
Current smoker	111 (11.1)
Former smoker	151 (15.1)
LVEF at index admission, %	
Mean (SD)	27.2 (6.0)
Median (range)	30 (5.0, 35.0)
BNP at index admission, pg/mL (*n* = 665)^b^	
Mean (SD)	1172.0 (1380.9)
Median (range)	800 (200, 12,568)
NT-proBNP at index admission, pg/mL (*n* = 303)	
Mean (SD)	4074.0 (6741.5)
Median (range)	1425 (500, 45,000)
NYHA functional status at or before index date,^a^ *n* (%)	
Class I	56 (5.6)
Class II	276 (27.5)
Class III	460 (45.9)
Class IV	127 (12.7)
Class not recorded	83 (8.3)
Weight at admission, kg (*n* = 806)	
Mean (SD)	89.8 (22.1)
Median (range)	87.9 (40.8, 202.6)
Height at admission, m (*n* = 730)	
Mean (SD)	1.7 (0.1)
Median (range)	1.7 (1.3, 2.0)
BMI at admission, kg/m^2^ (derived, *n* = 605)	
Mean (SD)	31.2 (7.1)
Median (range)	30.0 (16.3, 55.7)
Duration of follow-up, months^c^	
Mean (SD)	26.1 (13.7)
Median (range)	24.2 (0.1, 51.4)
Died before end of follow-up, *n* (%)	37 (3.7)
Died from HF or HF-related complications	24 (2.4)
Comorbidities/risk factors in ≥ 10% of patients, *n* (%)	
Hypertension	713 (71.2)
Hyperlipidemia	514 (51.3)
Myocardial infarction	333 (33.2)
Diabetes without end-organ damage	282 (28.1)
Diabetes with end-organ damage (retinopathy, neuropathy, nephropathy)	166 (16.6)
History of tobacco use/smoking	262 (26.2)
Chronic obstructive pulmonary disease	224 (22.4)
Peripheral vascular disease	210 (21.0)
Depression	209 (20.9)
Cerebrovascular disease	178 (17.8)
History of atrial fibrillation/flutter	120 (12.0)
Moderate to severe renal disease	113 (11.3)
Use of therapeutic devices before or at index date,^a^ *n* (%)	
ICD, CRT, or combination	227 (22.7)
Cardiac pacemaker	54 (5.4)

*BMI* body mass index, *BNP* brain natriuretic protein, *CRT* cardiac resynchronization therapy, *HF* heart failure, *ICD* implantable cardioverter defibrillator, *LVEF* left ventricular ejection fraction, *NT* N-terminal, *NYHA* New York Heart Association, *SD* standard deviation

^a^Index date defined as the date on which the patient was first hospitalized with a primary diagnosis of HF between 1 July 2013 and 30 June 2016.

^b^A total of 1 patient with BNP > 15,000 was recorded as missing.

^c^Follow-up duration calculated as number of months between the study index date and last available medical record or date of death, whichever is earliest.

### Treatment Patterns

Of the 1002 patients, 882 (88.0%) received HF-related medications at admission, during the admission, and/or during the discharge (Supplementary Table 2). The majority of patients (59.1%) received BBs at admission; the most common were carvedilol [mean total daily dose, 15.7 mg (SD 13.5)] or metoprolol [mean total daily dose, 53.5 mg (SD 33.7)]. The next most common drug classes received at admission were diuretics (57.4%) and ACE inhibitors (47.5%). The use of nearly all drug classes increased during hospitalization (Supplementary Table 2). Among patients receiving BBs, ACE inhibitors, or ARBs, dose modifications were most commonly dose increases (Table 2). Overall, 865 patients (86%) received oral and/or intravenous diuretics at any time during the index hospitalization, including at discharge.

**Table 2:** Dose modifications during hospital admission or at discharge and primary reasons for the modifications

Reason, *n* (%)	Beta blockers	Angiotensin-converting enzyme inhibitors	Angiotensin receptor blockers
Dose modification, *N*^a^	884	657	218
Dose decreased	27 (3.1)	22 (3.4)	6 (2.8)
Dose increased	375 (42.4)	261 (39.7)	72 (33.0)
Medication discontinued	42 (4.8)	32 (4.9)	10 (4.6)
Dose decreased or discontinued, then increased or restarted	19 (2.2)	8 (1.2)	6 (2.8)
No change	408 (46.2)	327 (49.8)	122 (56.0)
Data not available/don’t know	16 (1.8)	9 (1.4)	3 (1.4)
Primary reason for dose modification, *N*^b^	460	321	93
Hypotension	25 (5.4)	15 (4.7)	7 (7.5)
Bradycardia	12 (2.6)	3 (0.9)	0
Fatigue	15 (3.3)	2 (0.6)	1 (1.1)
Low cardiac output	14 (3.0)	6 (1.9)	1 (1.1)
Treatment with inotropic medications	3 (0.7)	5 (1.6)	1 (1.1)
Acute kidney injury	0	11 (3.4)	1 (1.1)
Other	11 (2.4)	14 (4.4)	3 (3.2)
Don’t know/unknown	7 (1.52)	7 (2.2)	2 (2.2)
Missing^c^	391 (85.0)	267 (83.2)	77 (82.8)

^a^Percentages based on *n*/*N*, where *N* is the total number of patients receiving specified treatment. Patients may have had more than one category of dose modification.

^b^Percentages based on *n*/*N*, where *N* is the total number of patients with a dose modification.

^c^The missing observations almost always were associated with the event when the dose was increased.

### HR Control

For the index hospitalization, patients’ mean HR at discharge was 73.5 bpm (SD 11.5). The mean HR control between admission and discharge was − 19.5 bpm (SD 17.2), representing an average reduction of 18.8% (Table 3). Overall, most patients achieved some degree of HR control between admission and discharge: 52.1% (*n* = 522) achieved ≥ 20% reduction, 38.4% (*n* = 385) achieved < 20% reduction, and 9.5% (*n* = 95) achieved no reduction. Histograms of HRs at admission and discharge and the reduction in HR between admission and discharge are presented in Supplementary Fig. 1.

**Table 3:** Change in heart rate and blood pressure between index admission and discharge

	All patients (*N* = 1002)			Patients treated with diuretic (*n* = 865)		
	At admission	At discharge	Unit change	At admission	At discharge	Unit change
Heart rate, bpm						
Mean (SD)	93.1 (18.9)	73.5 (11.5)	− 19.5 (17.2)	93.4 (18.9)	73.4 (11.4)	− 20.1 (16.9)
Reduction,^a^ %			− 18.8			− 19.4
Blood pressure, mmHg						
Systolic						
Patients with data, *n* (%)	878 (87.6)	880 (87.8)		775 (89.6)	775 (89.6)	
Mean (SD)	139.9 (32.1)	121.3 (15.6)	− 18.4 (26.0)	139.9 (32.7)	120.8 (15.4)	− 19.0 (26.8)
Reduction,^a^ %			− 9.8			− 9.9
Diastolic						
Patients with data, *n* (%)	878 (87.6)	880 (87.8)		775 (89.6)	775 (89.6)	
Mean (SD)	80.6 (18.5)	71.7 (10.4)	− 8.8 (15.9)	80.6 (18.7)	71.4 (10.2)	− 9.0 (16.1)
Reduction,^a^ %			− 6.6			− 6.9
Unknown, *n* (%)	124 (12.4)	122 (12.2)		90 (10.4)	90 (10.4)	

*bpm* beats per minute, *SD* standard deviation

^a^Percent reduction in mean variable between admission and discharge.

### Rehospitalization Rates and Association with HR Control

**Fig. 1:**
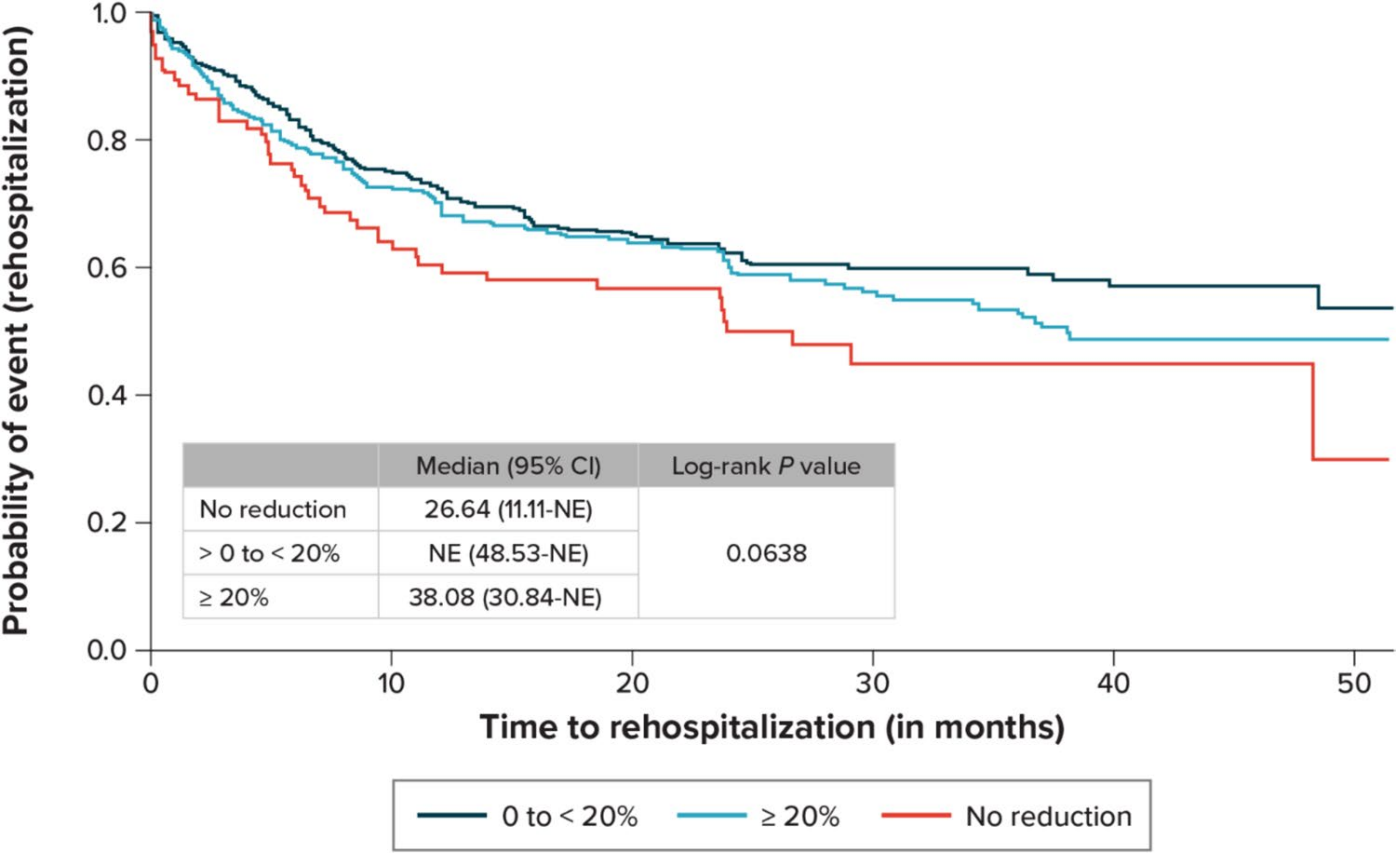
Kaplan–Meier analysis of time to rehospitalization stratified by change in heart rate during index admission for all patients.

Of the total study sample, 387 patients (38.6%) had at least 1 rehospitalization event, for a total of 674 rehospitalizations (Table 4). Most rehospitalizations (67.7%) were HF related. Approximately 14% of patients were rehospitalized within 30 days of discharge from index hospitalization. In the Kaplan–Meier analysis, the estimated median time to rehospitalization for all patients was 48.5 months. When examined by the degree of HR control, the time to rehospitalization tended to be longer for patients who experienced an HR reduction (versus those experiencing no HR reduction), although the difference was not statistically significant (Fig. 1).

**Table 4:** Rehospitalization after discharge from the index admission

	All patients (*N* = 1002)
Patients with ≥ 1 rehospitalization, *n* (%)	387 (38.6)
Patients with ≥ 1, 30-day rehospitalization,^a^ *n* (%)	53 (13.7)
Time to first rehospitalization, months	
Mean (SD)	8.9 (9.1)
Median (range)	6.0 (0, 48.5)
Rehospitalizations per patient, *n*	
Among all patients	
Mean (SD)	0.7 (1.1)
Median (range)	0 (0, 12)
Among patients with ≥ 1 rehospitalization	
Mean (SD)	1.7 (1.2)
Median (range)	1 (1, 12)
Primary reason for rehospitalization	
Total rehospitalizations, *n*	674
HF related, *n* (%)^b^	456 (67.7)
Other, *n* (%)^b^	218 (32.3)

^a^Percentage is *n*/*N*, where *N* is the total number of patients with any rehospitalization.

^b^Percentage is* n*/*N*, where *N* is the total number of rehospitalizations.

In the multivariable Cox regression analysis, HR control was found to be independently associated with a reduced risk of rehospitalization (Table 5). Compared with patients who had no HR reduction, those with an HR reduction of 0–20% had a 39% reduced risk of rehospitalization, with a hazard ratio of 0.61 (95% CI 0.43–0.85; *P* = 0.0041); for those with an HR reduction of ≥ 20%, the hazard ratio was 0.62 (95% CI 0.45–0.87; *P* = 0.0051) for a 38% decline in the risk. Among the covariates controlling for baseline characteristics, patients with a greater burden of comorbidity had a higher risk of rehospitalization [CCI score > 3 versus 0: hazard ratio 3.35 (95% CI 1.66–6.78)]. Likewise, patients with poorer functional status had elevated risk of rehospitalization compared with those with NYHA class II [NYHA class IV versus class II: hazard ratio 1.79 (95% CI 1.27–2.51); NYHA class III versus class II: hazard ratio 1.30 (95% CI 1.01–1.67)]. Patients who never smoked (versus former smokers) had a reduced risk of rehospitalization [hazard ratio 0.75 (95% CI 0.57–0.99)]. Patients’ age, sex, and insurance type were not correlated with risk for rehospitalization (Table 5). In the sensitivity analysis among patients with evidence of treatment with a diuretic at any time during the index hospitalization or discharge (*n* = 865), the associations were similar to those observed for the main analysis (Table 5).

**Table 5:** Factors associated with risk of rehospitalization, as assessed using Cox regression analysis, with the outcome variable of time to rehospitalization

Covariate	All patients			Patients treated with diuretic		
	Hazard ratio	95% CI	*P* value	Hazard ratio	95% CI	*P* value
HR control (versus no reduction)						
0% < reduction < 20%	***0.61***	**(*0.43–0.85*)**	***0.0041***	***0.72***	**(*0.49–1.06*)**	***0.0979***
≥ 20% reduction	***0.62***	**(*0.45–0.87*)**	***0.0051***	***0.69***	**(*0.47–1.00*)**	***0.0514***
Age group, years (versus ≥ 65)						
18–35	0.92	(0.41–2.07)	0.8345	0.72	(0.28–1.85)	0.4984
36–45	1.30	(0.88–1.92)	0.1915	1.18	(0.78–1.77)	0.4431
46–55	1.08	(0.80–1.47)	0.6143	1.03	(0.74–1.44)	0.8510
56–65	1.04	(0.80–1.35)	0.7650	1.03	(0.78–1.36)	0.8251
Female (versus male)	0.99	(0.80–1.23)	0.9133	1.00	(0.80–1.26)	0.9939
Insurance type (versus private)						
Medicare	1.03	(0.78–1.36)	0.8487	1.07	(0.79–1.45)	0.6589
Medicaid	1.17	(0.84–1.64)	0.3589	1.38	(0.96–1.97)	0.0788
Dual	1.28	(0.86–1.90)	0.2217	1.45	(0.95–2.20)	0.0824
Other	1.42	(0.96–2.12)	0.0827	**1.66**	**(1.09–2.51)**	**0.0177**
Smoking status (versus former smokers)						
Current smokers	0.95	(0.65–1.38)	0.7761	0.97	(0.66–1.43)	0.8850
Never smokers	***0.75***	**(*0.57–0.99*)**	***0.0436***	***0.72***	**(*0.54–0.96*)**	***0.0266***
CCI group (versus CCI = 0)						
CCI = 1	1.10	(0.52–2.32)	0.8104	1.00	(0.43–2.31)	0.9918
CCI = 2	1.93	(0.94–3.99)	0.0751	1.65	(0.73–3.69)	0.2276
CCI ≥ 3	**3.35**	**(1.66–6.78)**	**0.0008**	**2.86**	**(1.31–6.29)**	**0.0087**
NYHA class (versus NYHA class II)						
Class I	0.72	(0.38–1.37)	0.3195	0.75	(0.38–1.47)	0.4000
Class III	**1.30**	**(1.01–1.67)**	**0.0436**	**1.30**	**(0.99–1.70)**	**0.0550**
Class IV	**1.79**	**(1.27–2.51)**	**0.0008**	**1.57**	**(1.08–2.26)**	**0.0171**
Class not recorded	1.02	(0.66–1.59)	0.9148	1.03	(0.65–1.63)	0.8914
Therapeutic devices (versus none)						
Implantable cardioverter defibrillator	1.08	(0.81–1.43)	0.6181	1.06	(0.79–1.43)	0.6854
Other	0.96	(0.72–1.29)	0.7800	0.93	(0.67–1.27)	0.6328
LVEF at index admission (versus 30% to < 35%)						
< 20%	1.26	(0.87–1.81)	0.2178	1.09	(0.73–1.63)	0.6626
20% to < 25%	1.00	(0.73–1.36)	0.9982	0.95	(0.68–1.32)	0.7470
25% to < 30%	0.93	(0.72–1.19)	0.5583	0.96	(0.74–1.25)	0.7760
Any BB (versus no BB)	**1.79**	**(1.22–2.63)**	**0.0030**	**1.57**	**(1.02–2.42)**	**0.0392**
Any ivabradine (versus no ivabradine)	1.04	(0.63–1.73)	0.8781	1.10	(0.65–1.86)	0.7202

Bold indicates patients with significantly greater risk of rehospitalization; bold italics represents those with a significantly lower risk of rehospitalization

*BB* beta blocker, *CCI* Charlson Comorbidity Index, *CI* confidence interval, *HR* heart rate, *LVEF* left ventricular ejection fraction, *N/A* not applicable, *NYHA* New York Heart Association

## Discussion

We report findings from a large real-world study of patients with HFrEF in the USA and describe their treatment characteristics, clinical outcomes, and risk of rehospitalization. In our cohort of 1002 patients hospitalized for HFrEF, patients experienced a mean HR reduction of 19.5—equivalent to an 18.8% decline—between admission and discharge. Furthermore, our analysis demonstrates that patients who achieved HR control during the index hospitalization were at significantly reduced risk of rehospitalization over a median follow-up of more than 2 years, possibly owing to reduced cardiovascular morbidity associated with lower HR [13]; this effect was largely the same for both the < 20% (modest) and ≥ 20% (significant) HR control. Clinical practice guidelines have discussed the importance of HR targets for patients with HF and atrial fibrillation [17], and evidence from the Get With The Guidelines-Heart Failure program has suggested HRs between 70 bpm and 75 bpm to be optimal and associated with lowest risk of mortality [18]. Findings from the present study highlight the fact that HR control is an independent predictor of rehospitalization in HFrEF, regardless of the observed clinical parameters and pharmacotherapeutic use (including betablockers).

There are limited real-world data assessing the impact of HR control during acute HF hospitalization on the risk of rehospitalization and its associated prognostic factors in the HFrEF setting. The SHIFT trial using ivabradine also supports this notion of HR reduction as a clinical determinant of improved HF outcomes [10]. In clinical trials, ivabradine reduced HR and improved clinical outcomes in patients with HF, including those with HFrEF [10, 13, 14]. In the current study, only a few patients had received treatment with ivabradine. Our analysis also showed that, besides the effect of HR control, comorbidities and more severe functional disease increased the risk of rehospitalization, corroborating previous findings [19–23]. The all-cause rehospitalization rate at 30 days that we observed in this study was slightly lower than the previously reported rate of 20% [24]. Of note, in our analysis, it was interesting to observe that a meaningfully large proportion of patients (nearly 14%) did not initiate treatment with diuretics, possibly indicating a slight departure from the guideline recommendations [25]; this could also explain to some degree the absence of HR control during the index hospitalization in about 10% of the patients in our study.

There are limitations to our study. These patients represent a convenience sample that may not be generalizable to all patients with HFrEF, and the physicians who selected them may not be generalizable to all physicians who treat such patients. The potential for sampling bias by the physician was avoided using a pseudo-randomization approach to identify patients to be screened for potential inclusion in the study; however, some selection bias may still have been present. Information used in the analyses was restricted to data available in the patients’ medical records and may not be complete or entirely accurate. For example, abstracted prescription data did not contain the rationale for the drug or dose being prescribed. Further, if any patient had a medical encounter with another medical practitioner outside the provider network, that information may not have been recorded in their individual medical record held by the abstracting physician. Additionally, although a prescription may have been provided in the medical record, it is possible that the patient did not fill or take the prescribed medication. We collected data on a diverse array of potential prognostic factors, including NYHA functional class among other clinical characteristics, and these data were adjusted for in the multivariable analysis; however, the presence of some unobserved confounding cannot be ruled out. The HR target values vary between patients with HFrEF in sinus rhythm versus those with atrial fibrillation or atrial flutter. In our study, 12% of the patients had a history of atrial fibrillation or flutter; however, we did not separately examine HR control and associated risk of rehospitalization in this small subset of patients. A small proportion of patients (3.7%) died during the follow-up, which may present competing risk and could be associated with small bias in the estimation of precision. While our study demonstrates that HR reduction is associated with reduced risk of rehospitalization, we did not explore or identify the mechanism of benefit. Moreover, our analysis does not account for time-dependent risk factors that may influence rate of readmission, especially during the later part of the follow-up period. Our study also did not collect data on trajectory of HR changes over time during the course of hospitalization but only at admission and discharge. Additional studies to explore the mechanism of benefit from HR reduction, as well as to use an alternative approach to define exposure on the basis of trajectories of HR change, would further contribute to the available literature.

## Conclusions

This real-world analysis demonstrates that the reduction in HR between admission and discharge was associated, independently of BB and other baseline factors, with reduced risk for rehospitalization and longer time to hospitalization. These findings indicate that HR control should be an important goal in the management of patients hospitalized for HFrEF, in addition to achieving target doses of BB.

### Supplementary Information

Below is the link to the electronic supplementary material.

Supplementary file1 (PDF 254 kb)

## Declarations

### Data availability

Qualified researchers may request data from Amgen clinical studies. Complete details are available at the following: http://www.amgen.com/datasharing.

## Abbreviations

ACE: Angiotensin-converting enzyme
ARB: Angiotensin II receptor blockers
BP: Blood pressure
BB: Beta blocker
CCI: Charlson comorbidity index
CI: Confidence interval
HFrEF: Heart failure with reduced ejection fraction
HR: Heart rate
HF: Heart failure
IQR: Interquartile range
LVEF: Left ventricular ejection fraction
NE: Not estimable
NYHA: New York Heart Association
SD: Standard deviation
