# Supplementary material for: Exploring the Association Between Heart Rate Control and Rehospitalization: A Real-World Analysis of Patients Hospitalized with Heart Failure with Reduced Ejection Fraction
Source: Drugs Real World Outcomes. 2024 Aug 1;11(3):501–11. doi: 10.1007/s40801-024-00436-z (PMC11365870; doi:10.1007/s40801-024-00436-z)
Supplement: Supplementary file 1 — Supplementary file1 (PDF 254 kb) [file 40801_2024_436_MOESM1_ESM.pdf]

SUPPLEMENTARY MATERIAL

Journal: *Drugs – Real World Outcomes*

Exploring the Association Between Heart Rate Control and Rehospitalization: A Real-World Analysis of Patients Hospitalized With Heart Failure With Reduced Ejection Fraction

Freny Vaghaiwalla Mody, MD[a]; Ravi K. Goyal, PhD[b]; Mayank Ajmera, PhD[b]; Keith L. Davis, MA[b]; Alpesh N. Amin, MD[c]

[a]Division of Cardiology, Veterans Affairs Greater Los Angeles HCS, the Department of Medicine at Ronald Reagan University of California Medical Center (UCLA) and David Geffen School of Medicine at UCLA, 11301 Wilshire Blvd, Los Angeles, CA 90073, USA;
[b]RTI Health Solutions, 3040 East Cornwallis Road, Research Triangle Park, NC 27709-2194, USA; OrCID (Ravi K. Goyal): 0000-0003-1632-036X
[c]University of California, 101 The City Drive South, Building 26, Room 1000, Irvine, CA 92868, USA

Supplementary Figure 1. Histograms of HRs at Admission and Discharge and the Change in HR Between Admission and Discharge

(A) HR at admission:

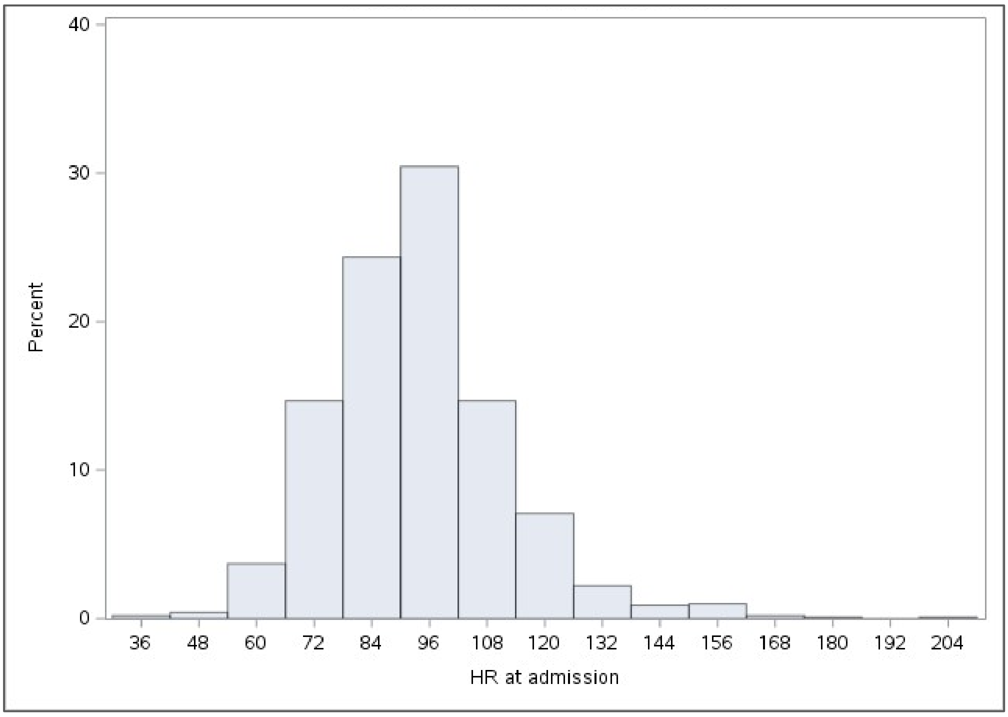

(B) HR at discharge:

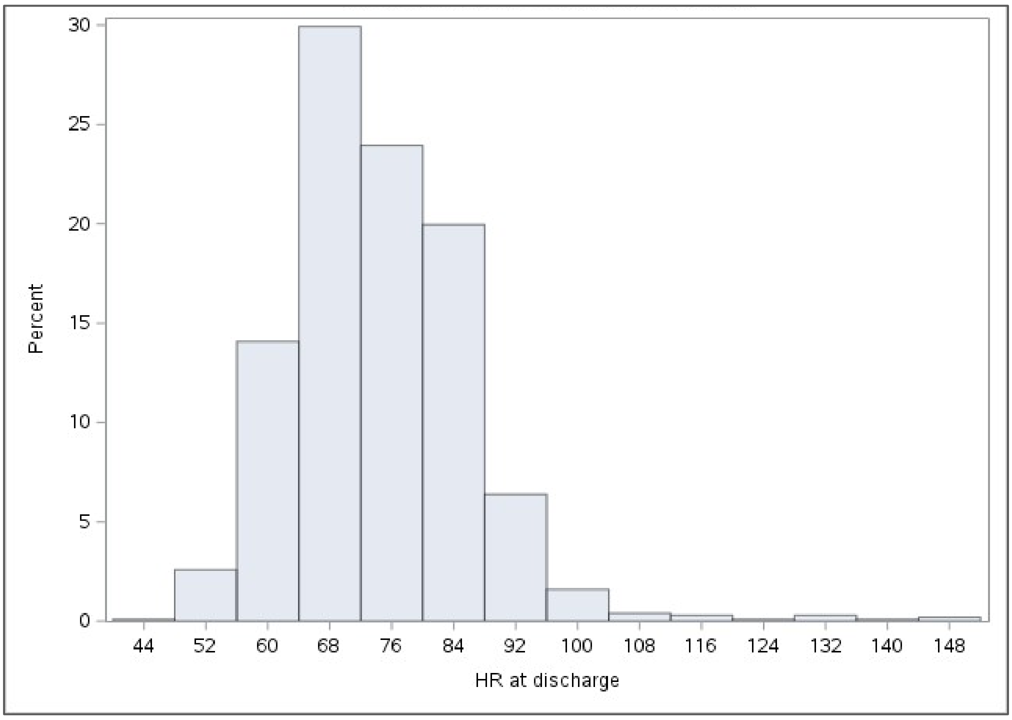

(C) Change in HR Between Admission and Discharge

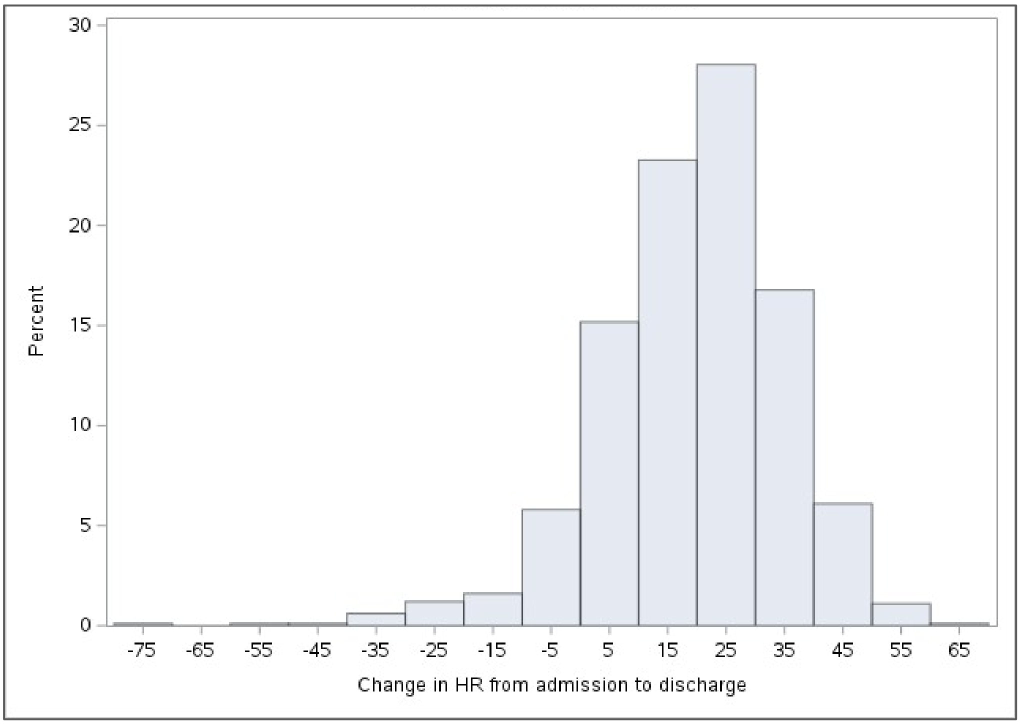

Supplementary Table 1. Physician Characteristics

Characteristic	Physicians (N=180)
Facility type, n (%)	
Teaching hospital	73 (40.6)
Nonteaching hospital	76 (42.2)
Freestanding community clinic (solo or group practice)	31(17.2)
Geographic region, n (%)	
Northeast	64 (35.6)
Midwest	32 (17.8)
South	55 (30.6)
West	29 (16.1)
Medical specialty, n (%)	
Cardiology	104 (57.8)
Internal medicine	15 (8.3)
Family medicine	18 (10.0)
Critical care	10 (5.6)
Emergency medicine	13 (7.2)
Geriatric medicine	7 (3.9)
Hospitalist	13 (7.2)
HF caseload in past month*	
Mean (SD)	79.3 (89.8)
Median (range)	50 (5, 500)
Years in practice treating patients with HF	
Mean (SD)	13.9 (7.1)
Median (range)	12 (3, 32)

HF=heart failure.

*Based on number of unique patients with HF treated in an inpatient setting.

Supplementary Table 2. Overview of Medications Prescribed in ≥5% of Any Time Period (N=1002)

Medication, n (%)	Immediately Before or at Time of Index Admission	During Course of Admission (Newly Initiated or Continued)	At Discharge (Newly Prescribed or Continued)
Beta blockers	592 (59.1)	767 (76.6)	715 (71.4)
Bisoprolol	26 (2.6)	21 (2.1)	29 (2.9)
Carvedilol	260 (26.0)	367 (36.6)	261 (26.1)
Metoprolol	256 (25.6)	358 (35.7)	396 (39.5)
ACE inhibitors	476 (47.5)	590 (58.9)	546 (54.5)
Enalapril	75 (7.5)	87 (8.7)	72 (7.2)
Lisinopril	340 (33.9)	430 (42.9)	418 (41.7)
ARBs	174 (17.4)	194 (19.4)	190 (19.0)
Losartan	119 (11.9)	129 (12.9)	120 (12.0)
Valsartan	40 (4.0)	53 (5.3)	52 (5.2)
Diuretics (oral or intravenous)	575 (57.4)	768 (76.7)	716 (71.5)
Mineralocorticoid receptor antagonist	142 (14.2)	295 (29.4)	355 (35.4)
Calcium channel blockers	120 (12.0)	84 (8.4)	82 (8.2)

Medication, n (%)	Immediately Before or at Time of Index Admission	During Course of Admission (Newly Initiated or Continued)	At Discharge (Newly Prescribed or Continued)
Other vasodilators	94 (9.4)	163 (16.3)	155 (15.5)
Antiarrhythmic/inotropic drugs	50 (5.0)	408 (40.7)	87 (8.7)
Sacubitril/valsartan	8 (0.8)	43 (4.3)	84 (8.4)
No medication	120 (12.0)	0	0
Don’t know	27 (2.7)	29 (2.9)	53 (5.3)

ACE=angiotensin-converting enzyme; ARB=angiotensin receptor blocker.
